# Gender-Specific Analyses of the Prevalence and Factors Associated with Substance Use and Misuse among Bosniak Adolescents

**DOI:** 10.3390/ijerph120606626

**Published:** 2015-06-10

**Authors:** Natasa Zenic, Admir Terzic, Jelena Rodek, Miodrag Spasic, Damir Sekulic

**Affiliations:** 1 Faculty of Kinesiology, University of Split, Teslina 6, Split-21000, Croatia; E-Mails: natasazenic@kifst.hr (N.Z.); jelena.rodek@kifst.hr (J.R.); miodrag.spasic@kifst.hr (M.S.); 2 High School “Hasan Kikic”, Sarajevks 1, Gradacac-76250, Bosnia and Herzegovina; E-Mail: admir.terza@bih.net.ba; 3 Faculty of Physical Education and Sport, University of Tuzla, 2^nd^ October 1, Tuzla-75000, Tuzla, Bosnia and Herzegovina; 4 Department of Health Care Studies, University of Split, R. Boskovica 31, Split-21000, Croatia

**Keywords:** substance misuse, puberty, academic achievement, parental monitoring, sport participation

## Abstract

Ethnicity and religion are known to be important factors associated with substance use and misuse (SUM). Ethnic Bosniaks, Muslims by religion, are the third largest ethnic group in the territory of the former Yugoslavia, but no study has examined SUM patterns among them. The aim of this study was to explore the prevalence of SUM and to examine scholastic-, familial- and sport-factors associated with SUM in adolescent Bosniaks from Bosnia-and-Herzegovina. The sample comprised 970 17-to-18-year-old adolescents (48% boys). Testing was performed using an previously validated questionnaire investigating socio-demographic-factors, scholastic-variables, and sport-factors, cigarette smoking, alcohol drinking, simultaneous smoking and drinking (multiple SUM), and the consumption of other drugs. The 30% of boys and 32% of girls smoke (OR = 1.13; 95% CI = 0.86–1.49), 41% of boys and 27% of girls are defined as harmful alcohol drinkers (OR = 1.94; 95% CI = 1.48–2.54), multiple SUM is prevalent in 17% of boys and 15% of girls (OR = 1.11; 95% CI = 0.79–1.56), while the consumption of other drugs, including sedatives, is higher in girls (6% and 15% for boys and girls, respectively; OR = 2.98; 95% CI = 1.89–4.70). Scholastic achievement is negatively associated with SUM. SUM is more prevalent in those girls who report higher income, and boys who report a worse familial financial situation. The study revealed more negative than positive associations between sport participation and SUM, especially among girls. Results can help public health authorities to develop more effective prevention campaign against SUM in adolescence.

## 1. Introduction

Substance use and misuse (SUM) includes cigarette smoking, alcohol drinking, other drug consumption (i.e. opiates, cannabinoids, prescription drugs) and other similar behaviors. Although mostly recognized as major public health problem, SUM is also linked with numerous other incidents such as aggression, traffic accidents, domestic violence, *etc.* [1,2,3]. Therefore, investigations have frequently tried to explore the factors associated with SUM [4,5,6]. It is accepted that those who have reached the age of 21 without consuming such substances will likely never engage in such behavior, while those who consume substances during adolescence are more likely to be addicted later in life [7,8].

Studies have frequently investigated factors related to SUM prevalence in adolescents while trying to identify those factors directly and indirectly related to SUM among adolescents in different socio-cultural environments [9]. More precisely, every society has its own specific norms, habits, laws, socio-cultural context and tradition, which directly or indirectly modulate habits and behaviors, and therefore directly or indirectly define SUM [10]. Consequently, protective- and risk-factors related to SUM should be explored within each socio-cultural context, as specifically as possible.

Ethnicity is one of the socio-cultural circumstances known to be related to SUM [11,12]. Previous studies indicate that different religions and ethnicities have varying attitudes to SUM [13,14,15]. It therefore seems reasonable to control this factor while investigating SUM patterns and correlates in adolescence. From our perspective, this is particularly interesting in those societies and countries where other socio-cultural circumstances (e.g., availability of education, sport, financial situation, *etc.*) are similar across different ethnicities.

Bosnia and Herzegovina is a country on the Balkan Peninsula, one of the former Yugoslav Republics, and home to three ethnic groups; Bosniaks (mostly Muslims), Serbs (mostly Christian Orthodox), and Croats (mostly Catholics). What is important for this country is the fact that all three ethnicities speak practically the same language, participate in the same scholastic system, and share a similar (*i.e.*, ex-Yugoslav) social and cultural background. At the bottom line, the key difference between the three main ethnicities is found in their religions and some religiously inherited traditional norms. While studies have so far examined SUM among Croatian (*i.e.*, Catholic) adolescents, no study has examined SUM templates and factors related to SUM solely in Bosniak adolescents (*i.e.*, Muslims) [16,17].

Studies examining adolescent SUM templates and correlates in the territory of former Yugoslavia are scarce. Further, practically all studies have so far examined adolescent Croats, and/or observed overall prevalence regardless of the ethnicity of the observed participants. Moreover, no study has specifically examined children from other ethnic groups in the territory. Apart from just one investigation that examined doping and SUM behavior in Muslim athletes, we also found no study that investigated SUM patterns and correlates in Muslims resident in the territory of former Yugoslavia [18]. Knowing that Muslims (mainly native Bosniaks) are the third largest ethnic group in the territory of the former Yugoslavia, the problem is even more pressing.

Policy and prevention strategies against SUM should rely on precise data for specific socio-cultural circumstances. Therefore, the first aim of this study was to explore the prevalence of SUM in Bosniak (Muslim) adolescents from Bosnia and Herzegovina. In addition, we examined socio-demographic, scholastic, familial and sport factors associated with SUM in the studied adolescents.

## 2. Materials and Methods

### 2.1. Participants

The sample comprised 970 17- to 18-year-old adolescents (48% boys) who were randomly selected from Tuzla Canton in Bosnia and Herzegovina. We specifically targeted this region since this is part of the country where there is no evident segregation between the three constitutive ethnicities (*i.e.*, Serbs, Bosniaks, and Croats). All participants were in their final grade of high school (*i.e.*, 4th grade of high school, or 12th year of education). Simple random sampling was used in this study. In the first phase of our sampling procedure, we selected by lottery half of the schools in the Tuzla Canton. In the next phase, one-third of all high school seniors were selected via lottery from the previously selected schools, resulting in a sample of 41 classes. The survey was administered on a single day, meaning that all high school seniors who were at school on that day were invited to participate. The response rate was over 99%. Although testing included more participants, for the purpose of this study we only examined those who declared themselves as ethnic Bosniaks, Muslim by religion. The sample of participants observed herein represents 23.8% of high-school-seniors (final graders) in the Canton, and 5% of high-school-aged children in the Canton for the observed school-year.

### 2.2. Variables

Testing was performed using an extensive self-administered questionnaire that consisted of the following four groups of variables: socio-demographic variables, scholastic variables, familial factors, and sport factors. Questionnaire was previously used on similar samples and found to be reliable and valid measuring tool [2].

Socio-demographic data: The socio-demographic variables in this study included age, gender, ethnicity and religion.

Scholastic variables: Participants were asked about their scholastic achievement (grade-point average) over the last two years (a five-point scale ranging from excellent to poor), unexcused school absence (number of non-excused absences measured in teaching hours; a five-point scale ranging from “less than 5 hours” to “20 hours or more”), behavioral grade (a five-point scale ranging from excellent to poor), and overall school absence (a four-point scale ranging from “almost never” to “often”).

Familial factors included four questions: (i) “How often do you have a conflict with your parents?”; (ii) “How often are your parents absent from home, including for their work obligations?”; (iii) “How often do your parents ask you questions about your friends, scholastic achievement, problems, and other personal issues?” (The participants responded on a four-point scale ranging from “almost never” to “regularly”); and (iv) “How would you rate how much your parents care about you and your personal life?” (The participants responded on a four-point scale that included “They do not care at all”, “They do not care enough”, “They are relatively concerned”, and “They are highly concerned”).

Sport factors: We assessed individual and team sport participation separately (both on a three-point scale: Yes; Yes, but I quit; No, never). In addition, participants were asked about the amount of time they spend in connection with sports and their sport competitive achievements using an ordinal questionnaire form (see additional Tables for details).

Substance misuse data included cigarette smoking, alcohol drinking, multiple (simultaneous) SUM and consumption of other drugs. Participants were asked about their smoking habits, alcohol consumption, and drug (*i.e.*, opiate) usage. Current smoking was tested on a six-point scale (“I have never smoked”—“Quit”—“Currently smoking from time to time, but not daily”—“I’m smoking daily less than 10 cigs”—“I’m smoking daily 10–20 cigs”—“I’m smoking more than a pack daily”). For the purpose of this study and statistical calculations (see below for details) the participants were observed as “non-smokers” and “smokers”. Alcohol consumption was measured using the AUDIT questionnaire. In this questionnaire, participants answer ten items and the scores for each item range from 0 to 4, which defines the hypothetical range of a minimum of 0 to maximum of 40. The results of AUDIT scale were later divided into “harmful drinking” (HD; scores of 11 or above) and “non-harmful drinking” (scores below 11), which allowed us to observe the results as a categorical variable [16,19]. Those participants who declared smoking and harmful alcohol drinking were categorized in simultaneous SUM. The sub-scale for drug consumption included questions about the consumption of marijuana, hashish, heroin, cocaine, most party drugs (e.g., ecstasy, amphetamines, and others), inhalants, and sedatives. A seven-point range of consumption was offered for each question (”Never”—“Ever tried (*i.e.*, only once)”—“More than once”—“3–5 times”—“6–9 times”—“20–39 times”—“40 and more”). Participants were further categorized as drug users or non-users (*i.e.*, drug users were all participants who declared they had used as least one substance more than once).

### 2.3. Ethics

Before the study, the complete procedure and aim of the testing were explained to all participants. All subjects gave their informed consent for inclusion before they participated in the study. The study was conducted in accordance with the Declaration of Helsinki, and the protocol was approved by the Ethics Committee of University of Split, Faculty of Kinesiology (Project identification code: 2181-205-02-05-14-005).Testing was strictly anonymous, meaning that no personal data were collected (e.g., date of birth, city of birth, or specific club or sport participation). Multiple-choice answers were offered where possible (see the Results section for more details). Testing occurred in a group of at least 12 examinees. Each examinee was told that the testing was strictly anonymous, that he/she could refuse to participate and that they could leave some questions and/or the entire questionnaire unanswered. When the testing was completed, each examinee placed form in the closed box. On the next day, the boxes were opened by an investigator who had not tested the participants.

### 2.4. Statistics

Statistics included counts (frequencies) and percentages. Differences between genders were established using the Kruskal-Wallis ANOVA (KW) and *t*-test for independent samples when appropriate. To define whether different genders have different outcomes on a particular SUM measure, the odds ratio (OR) with 95% confidence interval (95% CI) were calculated. The same statistics were calculated to determine the associations between different types of SUM (HD; cigarette smoking and other drug consumption).

Forward conditional logistic regressions were calculated for binomial SUM criteria (harmful-drinking *vs.* non-harmful-drinking; cigarette-smoking *vs.* non-smoking; drug consumption *vs.* non-consumption; multiple-SUM-consuming *vs.* non-consuming) with socio-demographic, familial, scholastic, and sport factors as predictors. After calculating the ORs, a strong interrelationship between types of SUM was confirmed for both genders (see Results for more details). Therefore, we did not include one type of SUM as a predictor for another type of SUM in the multiple logistic regression calculations. Analyses were stratified by gender. Statistical significance was set at *p* < 0.05 (95%), and Statsoft’s Statistica version 12 was used for all analyses.

## 3. Results

**Figure 1:**
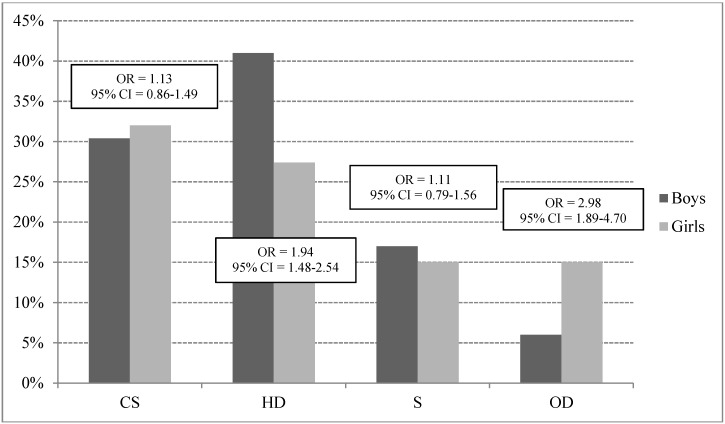
Comparison of cigarette smoking (CS), harmful drinking (HD), simultaneous smoking and harmful drinking (S) and consumption of other drugs (OD) by gender (OR—Odds Ratio; CI—confidence interval).

More than 30% of the boys and 32% of the girls smoke cigarettes, with no significant difference between genders in smoking prevalence (OR = 1.13; 95% CI = 0.86–1.49; *p* = 0.37). Harmful drinking is more prevalent in the boys (41%) than in the girls (27%) (OR = 1.94; 95% CI = 1.48–2.54; *p* < 0.01), with significant differences for total AUDIT score between genders (10.13 ± 8.67 and 7.45 ± 8.33; for boys and girls, respectively; t test = 4.91; *df* = 968; *p* < 0.05). Multiple SUM is prevalent in 17% of the boys and 15% of the girls, with no significant difference in the likelihood of such SUM between genders (OR = 1.11; 95% CI = 0.79–1.56; *p* = 0.54). The consumption of other drugs, including sedatives, is more prevalent in the girls than the boys (6% and 15% for boys and girls, respectively; OR = 2.98; 95% CI = 1.89–4.70; *p* < 0.01) (Figure 1).

The girls had better scholastic achievement (Supplementary Table S1), while the boys dominated in all observed sport factors (Supplementary Table S2). There were no significant gender differences in familial variables (Supplementary Table S3), while girls self-perceive higher levels of conflict with their parents, stronger parental care and more frequent parental questioning than the boys (Supplementary Table S4). Descriptive data stratified by gender and type of SUM are presented in Supplementary Tables S5–S12.

A higher prevalence of HD (OR = 2.5; 95% CI = 1.68–3.73; *p* < 0.01) and usage of other drugs (OR = 6.71; 95% CI = 2.88–15.63; *p* < 0.01) is found in those boys who smoke. Also, consumption of drugs is more prevalent in those boys who declared HD (OR = 3.74; 95% CI = 1.61–8.67; *p* < 0.01).

The odds of HD (OR = 4.65; 95% CI = 3.04–7.13; *p* < 0.01), and other drug use (OR = 4.27; 95% CI = 2.55–7.14; *p* < 0.01) increase with smoking in girls. Also, a prevalence of other-drug-consumption is higher in those girls who were defined as HD (OR = 12.09; 95% CI = 6.84–21.35; *p* < 0.05).

A higher prevalence of cigarette smoking is identified in those boys who are more absent from school (OR = 1.305; 95% CI = 1.004–1.695), with a poorer behavioral grade (OR = 1.854; 95% CI = 1.327–2.589), with a lower self-perceived family financial status (OR = 0.331; 95% CI = 0.156–0.699), and more frequent parental absence from home (OR = 1.251; 95% CI = 1.00–1.564) (Table 1). The model appropriately classified 72.3% of the participants.

Among the girls, cigarette smoking is related to poorer educational achievement (OR = 1.620; 95% CI = 1.303–2.014), frequent school absences (OR = 1.449; CI = 1.084–1.937), unexcused school absences (OR = 1.924; 95% CI = 1.427–2.594), longer participation in sports (1.428; OR = 1.119–1.821), but also to poorer sport achievement (OR = 0.692; 95% CI = 0.492–0.994), greater conflict with parents (OR = 1.456; 95% CI = 1.114–1.902) and frequent parental questioning (OR = 1.839; 95% CI = 1.332–2.540) (Table 1). In the final step, regression model successfully classified 73.8% of the girls.

**Table 1:** Forward conditional logistic regression results for the criterion—smoking incidence among boys and girls (OR—odds ratio; *p*—level of significance; CI—confidence interval).

Predictors	Boys			Girls	
	OR (*p*)	95% CI		OR (*p*)	95% CI
Grade point average				1.620 (0.01)	1.303–2.014
School absence	1.305 (0.04)	1.004–1.695		1.449 (0.02)	1.084–1.937
Unexcused absence				1.924 (0.01)	1.427–2.594
Behavioral grade	1.854 (0.01)	1.327–2.589			
Financial status	0.331 (0.01)	0.156–0.169			
Parental absence	1.251 (0.05)	1.001–1.564			
Conflict with parents				1.456 (0.01)	1.114–1.902
Parental questioning				1.839 (0.01)	1.332–2.540
Participation in sports				1.428 (0.01)	1.119–1.821
Sport achievement				0.692 (0.05)	0.492–0.994

Harmful alcohol drinking is more frequent among boys who achieved a lower grade-point average in the preceding school year (OR = 1.298; 95% CI = 1.047–1.608), who were more absent from school (OR = 1.664; 95% CI = 1.328–2.085), had a lower financial status of the family (OR = 0.495; 95% CI = 0.255–0.963) and a higher educational level of the mother (OR = 1.403; 95% CI = 1.079–1.823) (Table 2). In total, 62.2% of the participants were successfully classified.

The logistic regression model calculated for harmful alcohol drinking as a criterion variable successfully classified 80% of the girls. A higher prevalence of HD is evidenced for girls with a lower grade-point average (OR = 1.497; 95% CI = 1.195–1.876), with more unexcused absences (OR = 2.565; 95% CI = 1.848–3.560), who had achieved a better result in sports (OR = 1.822; 95% CI = 1.337–2.483), come from families with a better financial situation (OR = 2.519; 95% CI = 1.080–5.877), who perceive stronger parental care (OR = 2.254; 95% CI = 1.439–3.532), and are more frequently in conflict with their parents (OR = 1.690; 95% CI = 1.266–2.254) (Table 2).

**Table 2:** Forward conditional logistic regression results for the criterion—harmful alcohol drinking among boys and girls (OR—odds ratio; *p*—level of significance; CI—confidence interval)

Predictors	Boys			Girls	
	OR (*p*)	95% CI		OR (*p*)	95% CI
Grade point average	1.298 (0.02)	1.047–1.608		1.497 (0.01)	1.195–1.876
School absence	1.664 (0.01)	1.328–2.085			
Unexcused absence				2.565 (0.01)	1.848–3.560
Financial status	0.495 (0.04)	0.255–0.963		2.519 (0.04)	1.080–5.877
Parental care				2.254 (0.01)	1.439–3.532
Conflict with parents				1.690 (0.01)	1.266–2.254
Maternal education	1.403 (0.02)	1.079–1.823			
Sport achievement				1.822 (0.01)	1.337–2.483

Multiple SUM (simultaneous harmful drinking and smoking) is evidenced for those boys who were frequently absent from school (OR = 1.417; 95% CI = 1.034–1.941), had a poor behavioral grade (OR = 1.947; 95% CI = 1.379–2.747), a poorer family financial situation (OR = 0.340; 95% CI = 0.134–0.866), and who perceive lower parental care (OR = 0.693; 95% CI = 0.483–0.995), with 85% of the participants being successfully classified by logistic regression calculation (Table 3).

When calculated for simultaneous HD and smoking, the logistic regression revealed the higher prevalence of multiple SUM in those girls who had achieved a lower grade-point average (OR = 1.917; 95% CI = 1.475–2.492), were more absent from school (OR = 1.496; 95% CI = 1.034–2.164), had more unexcused school absences (OR = 1.854; 95% CI = 1.301–2.641), had self-reported an above-average financial situation (OR 3.913; 95% CI = 1.604–9.542), had more parental absences from home (OR = 1.435; 95% CI = 1.059–1.945), and had higher parental care (2.893; 95% CI = 1.607–5.208), with 88% of the participants being successfully classified (Table 3).

**Table 3:** Forward conditional logistic regression results for the criterion—simultaneous cigarette smoking and harmful drinking among boys and girls (OR—odds ratio; *p*—level of significance; CI—confidence interval).

Predictors	Boys			Girls	
	OR (*p*)	95% CI		OR (*p*)	95% CI
Grade point average				1.917 (0.01)	1.475–2.492
School absence	1.417 (0.03)	1.034–1.941		1.496 (0.04)	1.034–2.164
Unexcused absence				1.854 (0.01)	1.301–2.641
Behavior grade	1.947 (0.01)	1.379–2.747			
Financial status	0.340 (0.02)	0.134–0.866		3.913 (0.01)	1.604–9.542
Parental care	0.693 (0.05)	0.483–0.995		2.893 (0.01)	1.607–5.208
Parental absence				1.435 (0.03)	1.059–1.945

The higher prevalence of other drug consumption among the boys was related to higher achievement in sports (OR = 2.323; 95% CI = 1.417–3.807), greater conflict with parents (OR = 1.926; 95% CI = 1.250–2.967) and self-perceived lower parental care (OR = 0.474; 95% CI = 0.289–0.776). The logistic regression model successfully classified 94% of the subjects (Table 4).

Among girls, the consumption of other drugs is associated with greater school absences and unexcused school absences (OR = 1.710 and 1.612; 95% CI = 1.155–2.533 and 1.153–2.254), participation in individual sports (OR =0.499; 95% CI = 0.306–0.813), higher sport achievement (OR = 1.625; 95% CI = 1.142–2.313), a better financial situation of the family (OR = 8.059; CI = 2.904–22.365), the father’s advanced educational level (OR = 1.805; 95% CI = 1.234–2.640) and greater conflict with parents (OR = 2.125; 95% CI = 1.451–3.113) (Table 4). In total, 90% of the participants were successfully classified by logistic regression calculation.

**Table 4:** Forward conditional logistic regression results for the criterion—consumption of the other drugs among boys and girls (OR—odds ratio; *p*—level of significance; CI—confidence interval).

Predictors	Boys			Girls	
	OR (*p*)	95% CI		OR (*p*)	95% CI
School absence				1.710 (0.01)	1.155–2.533
Unexcused absence				1.612 (0.01)	1.153–2.254
Financial status				8.059 (0.01)	2.904–22.365
Paternal education				1.805 (0.01)	1.234–2.640
Parental conflict	1.926 (0.01)	1.250–2.967		2.125 (0.01)	1.451–3.113
Parental care	0.474 (0.02)	0.289–0.776			
Individual sports				0.499 (0.01)	0.306–0.813
Sport achievement	2.323 (0.03)	1.417–3.807			

## 4. Discussion

This study makes several important findings. First, SUM among adolescent Bosniaks is similar to those already reported for same-age adolescents of other ethnicities in the region. Next, scholastic achievement is strongly negatively related to SUM in both genders. Parental and familial factors are associated with SUM, but the studied variables have diverse relationships with different kinds of substances while the associations vary between boys and girls. Finally, sport factors are specifically related to SUM, but this is mostly evidenced for girls. These issues will be discussed below.

### 4.1. Prevalence of Substance Use and Misuse

In general, data on high rates of cigarette smoking are supportive to previous reports that found alarmingly high rates of such behavior on the territory of Bosnia and Herzegovina in adults, as well as in adolescents [1,17,20]. Also, the data on cigarette smoking for boys (30% are smokers) are consistent with results reported so far for the territory of former Yugoslavia [21]. But, to the best of our knowledge this is the first study to have examined the incidence of smoking exclusively among Muslim Bosniaks.

The prevalence of smoking among girls exceeds the figures so far reported for girls in the territory [17,21]. More precisely, the finding of 32% of Bosniak girls who reported smoking is 9% higher than the prevalence reported for Croatian girls although, according to the latest European School Survey Project on Alcohol and Other Drugs report, Croatia has one of the highest rates of adolescent smoking in Europe [22].

There are several possible reasons for such high rates of cigarette smoking in studied adolescents. First, smoking is socially accepted and allowed in public. Second, some parts of the Bosnia and Herzegovina are culturally oriented toward tobacco growing. Finally, cigarettes are relatively cheap (a pack rarely costs more than 3 USD), while minors can easily buy it (*i.e.*, there is no strict regulation against selling cigarettes to underage persons).

Previous studies indicated a high level of alcohol consumption in the region and our results support those reports [16]. Prior to this study, we had believed that the prevalence of such behavior in such participants (*i.e.*, Muslim Bosniak adolescents) would be lower than the prevalence reported previously for Croatian adolescents (*i.e.*, Catholics), mostly because of the known religious limits Islam imposes with regard to alcohol consumption [23,24]. Yet, adolescent Bosniaks consume alcohol similarly to their Croatian peers in the region [1,16].

Although somewhat surprising, this finding is in line with some previously published reports done on Muslim athletes [18]. The explanation provided in that study seems to also be appropriate for our results. Specifically, moderate alcohol drinking is common behavior among the majority of Muslims in this country, and is mostly influenced by the fact that Bosnia and Herzegovina is a multiethnic country.

Other drug consumption in the sample of Bosniak adolescents is not high, while girls are more likely to consume such substances. However, it must be stressed that such prevalence is related to the use of sedatives in girls. For the purpose of statistical analyses the self-reported consumption of any other drug (including prescription drugs like sedatives) is categorized as “consumption of other drugs”, which consequently boosted the prevalence of other drugs in the girls compared to the boys.

### 4.2. Scholastic Variables and Substance Use and Misuse

The results show a negative association between SUM and scholastic achievement in both the boys and girls. However, such a relationship is more evident in the girls than the boys. Studies so far suggest that there are causal influences between SUM and educational success, although there is no consensus on a causal effect. While some authors suggest that educational failure is actually a result of SUM due to the negative influence of SUM on cognitive functioning, others are more prone to the opinion that those children who have failed educationally are inclined toward socio-cultural circumstances in which substances are more likely to be consumed [25,26]. The latter considerations place poor scholastic performance within the context of adolescents’ rebellion against adult norms—including the norm against SUM.

In some cases, this association is explained by the “theory of problem behavior” [27]. According to this view, problem behaviors often appear in “tandem” (in this case, SUM and educational failure) partly because some people have a general tendency for such activities. In fact, it is claimed that a psychosocial tendency for unconventionality is a probable risk factor for both problems (scholastic failure and SUM).

While this study has no intention to explicitly define the cause-effect relationship between the variables of interest, we judged some issues as being of interest in that regard. It is interesting that the strongest associations are found between “school absences” and SUM. Therefore, we may suppose that misuse of substances (mainly cigarettes) leads to more frequent school absences. Of course, for a more profound analysis of the problem a longitudinal analysis is needed.

### 4.3. Familial Variables and Substance Use and Misuse

The association of SUM with perceived financial status of the family varies across samples that are observed [28,29]. In our study, lower financial status is associated with HD among boys, but HD is more frequent in girls who reported better family finances. Knowing the situation in the society where the sample was drawn from, we may propose the most probable reasons for such findings. In short, boys purchase alcohol cheaply in grocery stores and markets and consume it in different social circumstances (*i.e.*, beer is consumed even on the streets and in parks). At the same time, girls’ drinking is only socially accepted in bars and nightclubs, where alcohol is far more expensive.

Cigarette smoking is regularly reported as more prevalent in those children with a poorer socio-economic status [28]. But, we found no significant association between finances and smoking. Most probably, relatively low price of cigarettes is the main reason we failed to define a significant relationship between the children’s socio-economic status and cigarette smoking.

Our results show that the parental monitoring variables are stronger factors of influence on SUM for Bosniak girls than for boys. Girls who misuse substances report greater conflict with parents, but also regular parental questioning and strong parental care. For all studied substances, the associations are co-factored with poor scholastic achievement, indicating that those girls who misuse substances and have problems at school are at the same time in conflict with their parents. Therefore, either parents perceive the multiple problems of their daughters, or parental pressure has resulted in rebellion against traditional norms and consequently in SUM. For boys, parental variables are solely associated with the consumption of other drugs. Most likely, the social acceptance of smoking and drinking by males, together with the high prevalence of these types of SUM in the overall population, have resulted in the non-significant associations between the familial variables with cigarette smoking and alcohol drinking in boys.

### 4.4. Sport Factors and Substance Use and Misuse

Recent studies have pointed to the need for a more profound analysis of sport as a factor potentially related to SUM in adolescents as both a gender-specific and activity-specific factor of influence [16,17]. The results presented here support such observations. Namely, while being relatively unimportant in boys, sport factors are specifically associated with SUM in Bosniak girls.

The results indicate that longer participation in sports in the context of poor competitive achievement increases the likelihood of cigarette smoking in girls. It is possible that cigarette smoking has reduced the physiological capacities of young athletes and consequently altered their sport achievement [30]. On the other hand, we may also suppose that relatively longer sport participation but low competitive achievements have resulted in frustration and consequent SUM [31,32,33]. However, a longitudinal analysis of the studied factors is needed for a deeper analysis.

Although greater alcohol use in athletes has been shown before [30,33], this study goes further and demonstrates the specific effects with regard to competitive achievement (i.e. higher sport achievement in girls increases the likelihood of their harmful drinking). Studies have so far indicated the crucial role of post-sport social gatherings in explaining alcohol drinking among athletes [16,34]. It is well known that advanced competitive achievement is a factor which additionally contributes to the frequency and nature of such gatherings, which then result in higher alcohol consumption.

Our results showing the increased consumption of other drugs (mostly marihuana and sedatives) in girls who participate in individual sports while achieving a better competitive result are novel to some extent. More detailed analysis of the descriptive data reveals the true nature of such associations. Namely, 50% of those girls who reported misuse of other drugs are “former individual athletes”. Further analyses are needed to precisely explore the background of such association.

### 4.5. Study Limitations

This study focused solely on children of one ethnicity. However, since previous studies noted the need to control ethnicity and religion while studying SUM templates, we intentionally sampled only boys and girls who declared themselves as Bosniaks (*i.e.*, Muslims). One could argue that participants may lean toward socially acceptable answers when being tested. However, we believe that the fact that we studied high school seniors at the end of their academic year, the strict anonymity of the questionnaire, the study design, and our experience from previous testing reduced this possibility.

Also, for the purpose of meaningful comparison with previously reported data on the territory, this study investigated older adolescents (17–18 year old). On the other hand, it is known that children initiate with SUM during the course of adolescence. Therefore, further studies should explore SUM templates in younger participants.

Finally, the study was cross-sectional and hence we were unable to discuss cause-effect relationships between the observed variables. Therefore, although aware that this study is not the final word on the problem, we believe that the results and discussion contribute to the body of knowledge in this field.

## 5. Conclusions

Regardless of growing efforts aimed at preventing SUM across the whole territory, the prevalence of cigarette smoking in Bosniak adolescents (31% smokers) puts Bosnia and Herzegovina among those European countries with the highest rates of smoking among adolescents. Most probably, the lack of strong prohibiting policy, allowance of the smoking in the public-places, even at the closed- facilities, and low price of the cigarettes (*i.e.*, a pack rarely costs more than 3 USD), directly contribute to alarming prevalence of smoking in older adolescents in the country. The consumption of other substance (alcohol and other drugs) falls within the average range when compared to other European countries.

The results show a strong connection between scholastic achievements and SUM. However, different kinds of SUM are more negatively associated with academic results in girls than in boys. While having a lower income increases the risk of SUM in boys, those girls with an advanced socio-economic status are more prone to SUM.

The results extend previous knowledge on associations between sports and SUM by identifying the important role of competitive achievement in sports as a factor related to SUM in adolescence. In most cases, a higher competitive achievement is connected with increased SUM, and this is especially evidenced for girls. It is probable that girls who were once involved in sports may be trying to defy gender-based expectations and women’s stereotypical identity, which substance use might help them to accomplish.

Future studies should focus on longitudinal analyses of the problem while observing different ethnicities (*i.e.*, religions) simultaneously. It will allow more precise analysis of the SUM behaviors and factors of influence in adolescents.

## Supplementary Files

Supplementary File 1
