# Supplementary material for: Gender-Specific Analyses of the Prevalence and Factors Associated with Substance Use and Misuse among Bosniak Adolescents
Source: Int J Environ Res Public Health. 2015 Jun 10;12(6):6626–40. doi: 10.3390/ijerph120606626 (PMC4483720; doi:10.3390/ijerph120606626)
Supplement: Supplementary File 1 [file ijerph-12-06626-s001.pdf]

Supplementary Information

Gender-Specific Analyses of the Prevalence and Factors of Influence on Substance Abuse among Adolescent Bosniaks

Table S1. Scholastic factors by gender.

**Factors**	**Boys N;%**	**Girls N;%**	**Kruskal-Wallis (*p*)**
Grade point average			
Excellent	142;30	187;38	
Very good	176;37	199;41	
Good	138;29	65;13	
Sufficient	12;3	1;0	17.45
Failed	9;2	27;6	(0.01)
School absence			
Almost never	199;41	207;42	
Rarely	168;35	199;41	
From time to time	91;19	63;13	3.32
Often	27;6	15;3	(0.10)
Unexcused school absence			
<5 h	314;65	358;73	
6–10	97;20	81;17	
11–15	30;6	19;4	
16–20	15;3	7;1	12.19
>20	25;5	13;3	(0.01)
Behavioral grade			
Excellent	385;80	418;85	
Very good	57;12	43;9	
Good	30;6	17;3	
Sufficient	9;2	2;0	10.74
Failed	3;1	0;0	(0.01)

Table S2. Sport factors by gender.

**Factors**	**Boys N;%**	**Girls N;%**	**Kruskal-Wallis (*p*)**
Participation in individual sports			
Yes, I'm still participating	160;33	57;12	
Yes, but not anymore	193;40	176;36	86.79
No, never	131;27	252;51	(0.01)
Participation in team sports			
Yes, I'm still participating	164;34	59;12	
Yes, but not anymore	229;48	174;36	126.87
No, never	89;19	245;50	(0.01)
Time of the sports involvement			
Never involved	55;11	182;37	

Table S2. *Cont.*

**Factors**	**Boys N;%**	**Girls N;%**	**Kruskal-Wallis (*p*)**
<1 year	72;15	111;23	
1 to 5 years	172;36	102;21	118.19
>5 years	186;39	88;18	(0.01)
Competitive achievement in sport			
Never competed	169;35	294;60	
Lower ranks	271;56	131;27	
National level	30;6	35;7	39.74
National team	13;3	24;5	(0.01)

Table S3. Familial factors by gender.

**Factors**	**Boys N;%**	**Girls N;%**	**Kruskal-Wallis (*p*)**
Financial status			
Below average	12;3	2;0	
Average	431;90	448;91	0.47
Above average	37;8	34;7	(0.50)
Father's education			
Elementary	17;4	39;8	
High school	337;70	303;62	
College degree	58;12	70;14	0.185
University	65;14	65;13	(0.66)
Mother's education			
Elementary	104;22	136;28	
High school	308;64	234;48	
College degree	27;6	43;9	0.06
University	39;8	64;13	(0.77)

Table S4. Parental monitoring factors by gender.

**Factors**	**Boys N;%**	**Girls N;%**	**Kruskal-Wallis (*p*)**
Familiar conflict			
Almost never	196;41	130;27	
Rarely	185;39	218;44	
Often	82;17	104;21	20.19
Regularly	18;4	32;7	(0.01)
Parental absence			
Almost never	80;17	110;22	
Rarely	128;27	110;22	
Often	186;39	157;32	0.17
Regularly	85;18	107;22	(0.66)
Parental care			
They not care at all	9;2	8;2	

Table S4. *Cont.*

Factors	Boys N;%	Girls N;%	Kruskal-Wallis (*p*)
They do not care enough	14;3	10;2	
Relatively concerned	204;43	152;31	13.97
Highly	254;53	314;64	(0.01)
Parental questioning			
Almost never	18;4	10;2	
Rarely	60;13	59;12	
Often	209;44	133;27	23.07
Regularly	194;40	281;57	(0.01)

Table S5. Scholastic factors by type of substance abuse among boys (T–total sample, C–cigarette smokers, HD–harmful drinkers, S–simultaneous smokers and harmful drinkers, D–other drugs consumers).

Factors	T N;%	C N;%	HD N;%	S N;%	D N;%
Grade point average					
Excellent	142;30	35;25	42;21	19;23	8;29
Very good	176;37	43;31	80;40	24;30	9;32
Good	138;29	49;35	65;33	29;36	8;29
Sufficient	12;3	8;6	7;4	6;7	3;11
Failed	9;2	5;4	4;2	3;4	0;0
School absence					
Almost never	199;41	45;32	67;34	21;26	6;21
Rarely	168;35	48;34	65;33	25;31	7;25
From time to time	91;19	39;28	56;28	31;38	11;39
Often	27;6	12;9	15;8	6;7	4;14
Unexcused school absence					
<5 h	314;65	75;54	119;60	40;49	17;61
6–10	97;20	34;24	48;24	18;22	4;14
11–15	30;6	13;9	15;8	10;12	3;11
16–20	15;3	11;8	10;5	8;10	2;7
>20	25;5	11;8	9;5	7;9	2;7
Behavioral grade					
Excellent	385;80	93;66	146;74	49;60	21;75
Very good	57;12	25;18	32;16	16;20	2;7,1
Good	30;6	18;13	18;9	13;16	4;14
Sufficient	9;2	6;4	4;2	3;4	1;4
Failed	3;1	2;1	2;1	2;2	0;0

Table S6. Sport factors by type of substance abuse among boys (T—total sample, C—cigarette smokers, HD—harmful drinkers, S–simultaneous smokers and harmful drinkers, D—other drugs consumers).

**Factors**	**T N;%**	**C N;%**	**HD N;%**	**S N;%**	**D N;%**
Participation in individual sports					
Yes, I'm still participating	160;33	38;27	62;31	24;30	11;39
Yes, but not anymore	193;40	73;52	90;45	41;51	11;39
No, never	131;27	33;24	51;26	18;22	6;21
Participation in team sports					
Yes, I'm still participating	164;34	36;26	66;33	22;27	11;39
Yes, but not anymore	229;48	88;63	101;51	52;64	15;54
No, never	89;19	20;14	36;18	9;11	2;7
Time of the sports involvement					
Never involved	55;11	9;6	18;9	3;4	0;0
<1 year	72;15	22;16	31;16	13;16	2;7
1 to 5 years	172;36	65;46	83;42	40;49	9;32
>5 years	186;39	48;34	71;36	27;33	17;61
Competitive achievement in sport					
Never competed	169;35	43;31	62;31	21;26	3;11
Lower ranks	271;56	92;66	124;63	56;69	19;68
National level	30;6	6;4	10;5	3;4	2;7
National team	13;3	3;2	7;4	3;4	4;14

Table S7. Familial factors by type of substance abuse among boys (T—total sample, C—cigarette smokers, HD—harmful drinkers, S–simultaneous smokers and harmful drinkers, D—other drugs consumers).

**Factors**	**T N;%**	**C N;%**	**HD N;%**	**S N;%**	**D N;%**
Financial status					
Below average	12;3	4;3	8;4	4;5	2;7
Average	431;90	137;98	178;90	76;94	24;86
Above average	37;8	3;2	16;8	3;4	2;7
Father's education					
Elementary	17;4	9;6	10;5	7;9	1;4
High school	337;70	92;66	130;66	48;59	15;54
College degree	58;12	21;15	26;13	9;11	2;7
University	65;14	20;14	33;17	17;21	10;36
Mother's education					
Elementary	104;22	37;26	42;21	18;22	3;11
High school	308;64	87;62	125;63	51;63	19;68
College degree	27;6	8;6	13;7	6;7	2;7
University	39;8	12;9	23;12	8;10	4;14

Table S8. Parental monitoring factors by type of substance abuse among boys (T—total sample, C—cigarette smokers, HD—harmful drinkers, S—simultaneous smokers and harmful drinkers, D—other drugs consumers).

Factors	T N;%	C N;%	HD N;%	S N;%	D N;%
Familiar conflict					
Almost never	196;41	59;42	73;37	31;38	5;18
Rarely	185;39	49;35	78;39	30;37	11;39
Often	82;17	28;20	48;24	20;25	8;29
Regularly	18;4	8;6	4;2	2;2	4;14
Parental absence					
Almost never	80;17	20;14	31;16	11;14	3;11
Rarely	128;27	37;26	58;29	27;33	2;7
Often	186;39	51;36	69;35	24;30	15;54
Regularly	85;18	36;26	43;22	21;26	8;29
Parental care					
They not care at all	9;2	5;4	9;5	5;6	4;14
They do not care enough	14;3	9;6	8;4	6;7	2;7
Relatively concerned	204;43	55;39	76;38	30;37	11;39
Highly	254;53	75;54	110;56	42;52	11;39
Parental questioning					
Almost never	18;4	7;5	10;5	4;5	2;7
Rarely	60;13	17;12	21;11	8;10	4;14
Often	209;44	65;46	98;49	40;49	12;43
Regularly	194;40	55;39	74;37	31;38	10;36

Table S9. Scholastic factors by type of substance abuse among girls (T—total sample, C—cigarette smokers, HD—harmful drinkers, S—simultaneous smokers and harmful drinkers, D—other drugs consumers).

Factors	T N;%	C N;%	HD N;%	S N;%	D N;%
Grade point average					
Excellent	187;38	36;23	38;29	14;19	25;33
Very good	199;41	72;46	48;37	31;41	45;60
Good	65;13	30;19	28;22	16;21	5;7
Sufficient	1;0	0;0	1;1	0;0	0;0
Failed	27;6	18;12	15;12	14;19	0;0
School absence					
Almost never	207;42	47;30	48;37	17;23	21;28
Rarely	199;41	73;47	52;40	36;48	25;33
From time to time	63;13	31;20	26;20	18;24	24;32
Often	15;3	6;4	5;4	5;7	5;7
Unexcused school absence					
<5 h	358;73	86;55	73;56	36;48	40;53
6–10	81;17	44;28	29;22	20;27	10;13

Table S9. *Cont.*

Factors	T N;%	C N;%	HD N;%	S N;%	D N;%
11–15	19;4	17;11	11;8	11;15	9;12
16–20	7;1	5;3	5;4	4;5	4;5
>20	13;3	5;3	13;10	5;7	12;16
Behavioral grade					
Excellent	418;85	118;76	94;72	49;65	51;68
Very good	43;9	25;16	24;18	18;24	16;21
Good	17;3	13;8	13;10	9;12	8;11
Sufficient	2;0	1;1	0;0	0;0	0;0
Failed	0;0	0;0	0;0	0;0	0;0

Table S10. Sport factors by type of substance abuse among girls (T—total sample, C—cigarette smokers, HD—harmful drinkers, S—simultaneous smokers and harmful drinkers, D—other drugs consumers).

Factors	T N;%	C N;%	HD N;%	S N;%	D N;%
Participation in individual sports					
Yes, I'm still participating	57;12	20;13	12;9	8;11	18;24
Yes, but not anymore	176;36	59;38	57;44	30;40	37;49
No, never	252;51	78;50	62;48	38;51	20;27
Participation in team sports					
Yes, I'm still participating	59;12	22;14	18;14	7;9	23;31
Yes, but not anymore	174;36	67;43	44;34	29;39	25;33
No, never	245;50	67;43	68;52	39;52	27;36
Time of the sports involvement					
Never involved	182;37	45;29	49;38	24;32	21;28
<1 year	111;23	41;26	24;18	19;25	13;17
1 to 5 years	102;21	34;22	32;25	16;21	15;20
>5 years	88;18	37;24	25;19	17;23	26;35
Competitive achievement in sport					
Never competed	294;60	84;54	64;49	40;53	24;32
Lower ranks	131;27	60;38	37;28	28;37	25;33
National level	35;7	9;6	21;16	8;11	10;13
National team	24;5	4;3	8;6	;0	16;21

Table S11. Familial factors by type of substance abuse (T—total sample, C—cigarette smokers, HD—harmful drinkers, S—simultaneous smokers and harmful drinkers, D—other drugs consumers).

**Factors**	**T N;%**	**C N;%**	**HD N;%**	**S N;%**	**D N;%**
Financial status					
Below average	2;0	0;0	0;0	0;0	0;0
Average	448;91	138;88	113;87	59;79	53;71
Above average	34;7	19;12	18;14	17;23	22;29
Father's education					
Elementary	39;8	9;6	11;8	4;5	0;0
High school	303;62	94;60	70;54	46;61	31;41
College degree	70;14	29;19	20;15	6;8	14;19
University	65;13	25;16	30;23	20;27	30;40
Mother's education					
Elementary	136;28	35;22	26;20	12;16	5;7
High school	234;48	76;49	64;49	42;56	29;39
College degree	43;9	26;17	13;10	8;11	14;19
University	64;13	20;13	28;22	14;19	27;36

Table S12. Parental monitoring factors by type of substance abuse (T—total sample, C—cigarette smokers, HD—harmful drinkers, S—simultaneous smokers and harmful drinkers, D—other drugs consumers).

**Factors**	**T N;%**	**C N;%**	**HD N;%**	**S N;%**	**D N;%**
Familiar conflict					
Almost never	130;27	29;19	24;18	17;23	10;13
Rarely	218;44	70;45	54;42	33;44	29;39
Often	104;21	41;26	32;25	13;17	17;23
Regularly	32;7	17;11	21;16	13;17	19;25
Parental absence					
Almost never	110;22	26;17	23;18	13;17	3;4
Rarely	110;22	36;23	26;20	12;16	15;20
Often	157;32	52;33	40;31	24;32	16;21
Regularly	107;22	43;28	42;32	27;36	41;55
Parental care					
They not care at all	8;2	0;0	8;6	0;0	8;11
They do not care enough	10;2	7;4	1;1	1;1	1;1
Relatively concerned	152;31	47;30	30;23	19;25	20;27
Highly	314;64	103;66	92;71	56;75	46;61
Parental questioning					
Almost never	10;2	3;2	5;4	1;1	5;7
Rarely	59;12	16;10	19;15	7;9	14;19
Often	133;27	29;19	27;21	18;24	16;21
Regularly	281;57	109;70	80;62	50;67	40;53

© 2015 by the authors; licensee MDPI, Basel, Switzerland. This article is an open access article distributed under the terms and conditions of the Creative Commons Attribution license (http://creativecommons.org/licenses/by/4.0/).
